# Feasibility of transcranial magnetic stimulation adjuvant therapy for chronic respiratory diseases: a narrative review

**DOI:** 10.3389/fresc.2026.1668986

**Published:** 2026-04-29

**Authors:** Rui Xu, Geyi Wen, Yuhong Wang, Xiaoyun Wang, Yuanyuan Luo, Lili Yang, Xuesong Gai, Li Li

**Affiliations:** 1 Department of Rehabilitation Medicine, The First People’s Hospital of Yunnan Province, Affiliated Hospital of Kunming University of Science and Technology, Kunming, Yunnan, China; 2 Department of Pulmonary and Critical Care Medicine, The First People’s Hospital of Yunnan Province, Affiliated Hospital of Kunming University of Science and Technology, Kunming, Yunnan, China; 3 Department of Emergency Trauma Surgery, The First People’s Hospital of Yunnan Province. Affiliated Hospital of Kunming University of Science and Technology, Kunming, Yunnan, China

**Keywords:** transcranial magnetic stimulation, chronic respiratory diseases, pulmonary rehabilitation, respiratory dysfunction, neuromodulation

## Abstract

Chronic respiratory disorders (CRDs) are the third most common cause of cancer- and cardiovascular disease-related mortalities. Non-pharmacological interventions for CRDs effectively slow disease progression, improve quality of life, ameliorate symptoms, and potentially extend survival. Pulmonary rehabilitation faces limitations in respiratory training efficacy owing to methodological constraints, poor patient adherence, and questions about long-term benefits. Transcranial magnetic stimulation (TMS) has been used to demonstrate the capacity of cortical excitability modulation to cause neuronal plasticity. For respiratory function, TMS exhibits dual diagnostic and therapeutic applications; it can identify neural origins of diaphragmatic dysfunction, directly stimulate pathways to activate the diaphragm, and address comorbid anxiety and depression through limbic cortical circuitry. This review outlines the physiological respiratory mechanisms and evaluates the current evidence for TMS interventions in respiratory dysfunction. Our objectives are to elucidate TMS mechanisms in CRDs, evaluate its feasibility as an adjunctive respiratory training approach, and propose novel conceptual frameworks for respiratory impairment management.

## 1 Introduction

Chronic respiratory diseases (CRDs) encompass a spectrum of prevalent and persistent conditions that impair the airways and other structures of the lungs, which are the third leading cause of mortality globally following cardiovascular disease and cancer (1). Recognized by the World Health Organization as one of the four major categories of chronic human diseases, CRDs account for approximately 7.5 million deaths annually, constituting approximately 14% of global mortality (2). Conditions frequently discussed under the umbrella of respiratory diseases include chronic obstructive pulmonary disease (COPD), asthma, as well as major public health challenges like lung cancer, tuberculosis, and respiratory infections (3). Characteristic symptoms such as dyspnea, cough, fatigue, anxiety, and depression, often progressively worsen with disease advancement. For individuals with CRDs, non-pharmacological interventions have been demonstrated to attenuate disease progression, enhance quality of life, alleviate symptoms, and potentially improve survival (4). These interventions include smoking cessation (5), pulmonary rehabilitation (PR), oxygen therapy, nutritional management, self-management education, noninvasive ventilation, and psychosocial support (6). PR has demonstrated substantial efficacy in improving exercise capacity, muscle function, and symptom management, making it a strongly recommended therapeutic approach for various CRDs, especially COPD (7–9).

However, despite substantial evidence supporting PR as a cornerstone therapy for CRDs, significant limitations persist and warrant attention (10, 11). Currently, the respiratory training modalities available within the PR for patients with CRDs remain methodologically constrained, primarily consisting of direct inspiratory muscle training and indirect training through aerobic exercise (12–14). These techniques require active patient engagement and sustained adherence for 4–6 weeks to achieve meaningful physiological adaptation (15). However, many patients struggle to complete the full intervention without direct clinical supervision, and post-discharge compliance remains suboptimal (16). Dyspnea, the primary and most debilitating symptom in CRDs, has multifactorial etiologies, including complex pathophysiological alterations in the airways, lung parenchyma, gas exchange, cardiovascular function, respiratory muscle performance, and neuromodulatory mechanisms (17, 18). Critically, dyspnea represents a multidimensional subjective experience profoundly influenced by sensory, physical, and affective factors (19), complicating treatment approaches that focus solely on physiological parameters. Consequently, given the complex, multifactorial nature of dyspnea and the limitations of current therapeutic modalities, a critical question emerges: Are novel approaches capable of addressing or ameliorating breathlessness through multimodal intervention strategies?

With the rapid advancement of technology and neuroscience, research into the use of neuromodulation for studying and treating neurological pathologies is increasingly prevalent. Neuromodulation can be achieved through multiple approaches, including invasive and non-invasive methods. Most patients do not undergo invasive procedures, whereas non-invasive approaches garner greater attention (20, 21). Barker et al. (22) introduced the use of TMS in 1985. Based on the principles of electromagnetic induction and conversion, TMS produces a magnetic field via transient current in stimulating coil that enters the skull, generating induced current that stimulates neurons and triggers physiological and biochemical reactions (23, 24). As a noninvasive stimulation technique, TMS demonstrates excellent safety and tolerability (epilepsy incidence rate, <0.01%) (25), which has driven its widespread adoption across cognitive neuroscience, physiology, and clinical practice (26). Several TMS devices have been granted approval by the US Food and Drug Administration (FDA) (27). Organisations in several European countries have already addressed the evidence status of TMS efficacy in their depression management guidelines, endorsing its safety and clinical efficacy (28, 29). An increasing number of countries worldwide are adopting TMS therapy in clinical practice as a non-pharmacological treatment alternative for psychiatry, neurology and rehabilitation (26, 29–31).

Over the past decade, noninvasive brain stimulation applications have rapidly expanded (23) in studying brain–behavioral relationships, and treating various neurological and mental disorders has rapidly increased. It can regulate specific cortical–subcortical networks, enabling the controlled manipulation of behavior (32) and producing specific behavioral effects by releasing neurotransmitters into specific neural networks (33–36). TMS has emerged as a prominent area of research and has been used to treat stroke sequelae (32, 37–39), spinal cord injury (SCI) (40–42), multiple sclerosis（MS） (43–45), Alzheimer's disease（AD） (46, 47), emotional disorders (48–50), autism (51, 52) and sleep disorders (53, 54). The current guidelines recommend TMS as class I evidence for depression, neuropathic pain, and subacute stroke and class II evidence for Parkinson's disease, chronic stroke, and non-neuropathic pain (31). Furthermore, TMS serves as a recognized tool for studying respiratory corticospinal pathways, excitability, and inhibitory functions (55–61), and it can be utilized to evaluate the excitability of respiratory motor outputs (e.g., respiratory pathways, phrenic nerve, diaphragm) in various pathologies, including neurological and respiratory diseases like cerebral ischemia, SIC, and COPD (62–64). The sustained modulatory effects of TMS on cortical excitability form the basis of its application as a respiratory intervention (65–67). This is exemplified by its ability to improve respiratory function in neurological disorders through enhanced excitability of the phrenic nerve pathway and subsequent diaphragmatic contraction (68–71). Given this underlying mechanism, TMS may also hold therapeutic potential for CDRs characterized by diaphragmatic dysfunction. This paper systematically reviews the mechanism of action of TMS within the respiratory system, analyzes its therapeutic benefits, explores its potential in managing chronic respiratory diseases, and offers recommendations for clinical translation.

## 2 Potential mechanism of TMS action in CRDs

### 2.1 Respiratory physiology

Respiratory control is hierarchically organized into distinct levels. Automatic control is mediated by the brainstem via bulbospinal pathways, whereas voluntary control is primarily commanded by the cerebral cortex via the corticospinal tracts, with the brain serving as the central regulator (72). Respiratory activity is orchestrated by medullary respiratory rhythm generators. This rhythm is then modified by various lower brainstem nuclei, integrated into a coordinated signal, and ultimately converted into motor output through premotor efferent networks in the brainstem and spinal cord (73, 74).

**Figure 1:**
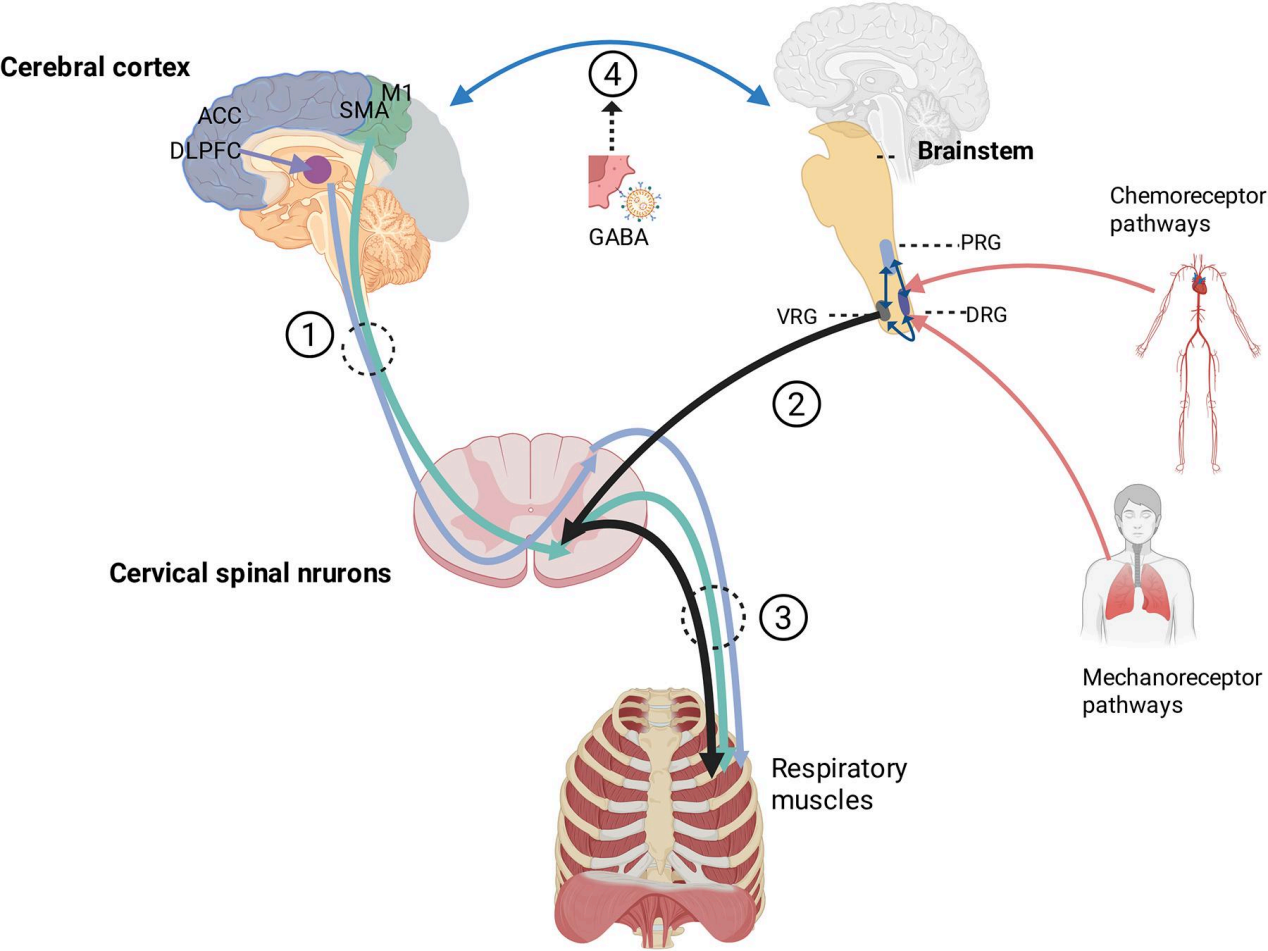
Schematic diagram of the neural pathways regulating voluntary and involuntary breathing. The illustration depicts a dual-pathway model for respiratory control. (1) Voluntary Pathway: Descending inputs from the primary motor cortex (M1) and supplementary motor area (SMA) project directly to cervical spinal motoneurons to drive respiratory muscles. Emotional states, processed by the dorsolateral prefrontal cortex (DLPFC), influence brainstem respiratory centers via limbic structures such as the amygdala and hypothalamus, leading to increased or irregular breathing. (2) Involuntary (Automatic) Pathway: The core pontomedullary rhythmogenic circuit, comprising the pontine respiratory group (PRG), ventral respiratory group (VRG), and dorsal respiratory group (DRG), generates and coordinates the respiratory rhythm. This circuit integrates feedback from chemoreceptor pathways (sensing blood O₂, CO₂, and pH levels) and mechanoreceptor pathways to drive the diaphragm and intercostal muscle. (3) Spinal Motor Output: The spinal motoneuron pool integrates supraspinal commands and transmits rhythmic output to the diaphragm via the phrenic nerve and to intercostal muscles via intercostal nerves. (4) Coordination Mechanism: Voluntary and involuntary pathways interact within the pontomedullary network. For instance, activation of dorsal anterior cingulate cortex (dACC) neurons recruits GABAergic inhibitory neurons in the pontine reticular nucleus (PnC), which subsequently suppress inspiratory activity in the ventrolateral medulla (VLM). This neural circuit results in prolonged expiration, reduced respiratory frequency, and concomitant alleviation of anxiety. Created in BioRender. XU, R. (2025) https://BioRender.com/ieox1xv.

In brief, the hierarchical structure of respiratory control comprises four principal levels (Figure 1):

Voluntary respiration (cortical level): This level is primarily regulated by the primary motor cortex (M1), receiving significant inputs from the supplementary motor area (SMA), the prefrontal cortex (PFC) and the anterior cingulate cortex (ACC). Descending commands travel via the corticospinal tracts to the respiratory motoneurons in the brainstem and spinal cord, governing respiration during volitional acts and stress responses (75, 76).

Involuntary respiration (brainstem level): This fundamental level is governed by the brainstem respiratory center, specifically the ventral respiratory group (VRG) and the dorsal respiratory group (DRG) within the medulla oblongata. These nuclei generate the essential respiratory rhythm and coordinate bulbospinal tract conduction (73).

Spinal conduction level: This level involves spinal motoneuron pools that relay commands via the phrenic nerve (C3–C5) and intercostal nerves (T1–T11).

Effector level: At the effector level, respiratory muscles—primarily the diaphragm—contract in response to neural drive (75, 76). Notably, respiratory motoneurons exhibit unique properties compared with other spinal motoneurons, integrating descending inputs from both the bulbospinal pathways (automatic ventilatory commands) and corticospinal pathways (voluntary commands) (76, 77). Furthermore, phrenic motoneurons receive corticobulbospinal motor inputs from limbic structures (78), which mediate the emotional modulation of breathing patterns (79–81).

### 2.2 TMS acts on the cortex

The rationale for targeting respiratory muscles (e.g., the diaphragm and intercostal muscles) with TMS primarily involves the activation of corticospinal pathways and the induction of neuroplasticity (82). Repetitive TMS (rTMS) noninvasively delivers electromagnetic pulses to cortical areas of the respiratory network, including the motor cortex (59, 83, 84), cortex (85, 86), and SMA (85, 87) via electromagnetic pulses. In response to this stimulation, neuronal membrane potentials are changed, and synaptic plasticity [long-term potentiation [LTP] or suppression of long-term depression [LTD]] is induced, which in turn modifies excitability inside respiratory regulatory neural networks. High-frequency rTMS enhances cortico-phrenic pathway excitability, improving the central drive to respiratory muscles and consequently augmenting respiratory muscle efficiency and endurance (88). Specifically, by stimulating the motor cortex, TMS activates corticospinal pathways that descend to synapse with phrenic motor neurons (PMNs) in the spinal cord, facilitating their excitability and ultimately enhancing respiratory muscle performance (89, 90). Collectively, these TMS-induced increases in pathway excitability likely augment diaphragmatic mobility and function.

A critical consideration is whether distinct cortical stimulation targets exert comparable effects on the diaphragmatic function. Initial investigations on respiratory TMS predominantly targeted the M1 (Brodmann area 4), which governs volitional breathing. Murphy et al. (91) first employed non-focal TMS to identify the optimal site for eliciting diaphragmatic motor-evoked potentials (DiMEPs), localized at approximately 2 cm anterior to the vertex along the sagittal midline, where bilateral diaphragmatic responses were consistently evoked. Subsequent research has increasingly focused on the SMA (Brodmann area 6), which regulates volitional respiratory behaviors, such as speech, coughing, and exercise-induced breathing adjustments. Maskill et al. (56) utilized a figure-of-eight coil positioned over the M1 to evoke contralateral diaphragmatic responses. Sharshar et al. (89) further demonstrated DiMEPs induction following TMS over the SMA anterior to the diaphragmatic M1. Accumulating evidence has identified both the SMA and M1 as key contributors to cortical respiratory regulation. Neuroimaging studies have consistently revealed SMA-M1 coactivation during human respiratory tasks, particularly volitional breathing (92). This functional integration arises from direct SMA–M1 connectivity via anterior corpus callosum fibers, which subserve bilateral motor coordination (93, 94). This neuroanatomical framework enables the coordinated bilateral control of the respiratory muscles, wherein the SMA orchestrates bilateral M1 output to the diaphragm and intercostal muscles, ensuring symmetrical contraction. Disruption of this circuit may manifest as motor apraxia or respiratory discoordination (71).

Building upon the established SMA–M1 connectivity, Raux et al. (95) demonstrated that high-frequency rTMS applied to the SMA enhanced diaphragmatic corticospinal excitability in healthy participants. Conversely, low-frequency rTMS over the lateral premotor cortex reduces corticospinal excitability, whereas identical stimulation parameters applied to the SMA produce no significant changes in diaphragmatic excitability. This suggests that high-frequency rTMS potentiates synaptic transmission and neuroplasticity, potentially augmenting diaphragmatic control by facilitating SMA connections with downstream respiratory networks (e.g., spinal PMNs) (73). This mechanism underpins the therapeutic potential of high-frequency rTMS for respiratory muscle weakness. By contrast, low-frequency rTMS targeting the lateral premotor cortex modulates the primary motor cortex or spinal pathways, thereby inhibiting diaphragmatic control. This modality shows promise in alleviating pathological respiratory hyperactivation, including dyspnea or spasmodic breathing. Notably, SMA pathways exhibit relative resistance to low-frequency inhibitory effects. Supporting this model, Raux et al. (96) identified SMA-mediated respiratory braking evokes physiological responses that correlate with unpleasant breathing sensations. These findings provide a neurophysiological rationale for exploring rTMS-based dyspnea relief, similar to established analgesic protocols.

### 2.3 TMS acts on the spinal cord

Within the neural circuitry governing volitional breathing, respiratory commands originate in the cortex and descend via corticospinal pathways. Although TMS targets cortical initiation sites, complementary stimulation can be applied at the cervical spinal level, leveraging the anatomical concentration of PMNs in the C3–C5 spinal segments that control diaphragmatic contraction (97). Lee et al. (64) demonstrated that cervical TMS effectively assessed diaphragmatic motor output excitability post-injury in rodent models, with laterally directed stimulation yielding optimal DiMEPs elicitation. Subsequent work by Lee et al. (98) systematically evaluated the effects of coil positioning (cranial, mid-thoracic, and caudal spinal levels) on DiMEPs generation in cervical SCI models. Their findings revealed that caudal stimulation generated significantly higher DiMEPs amplitudes than cranial approaches, although cervical contusion injuries maintained a diminished inspiratory drive despite normalized DiMEPs after magnetic stimulation. Importantly, given the known interspecies variations in respiratory neurophysiology, these rodent findings require validation through focused clinical investigations before their translational application in human patients. In a clinical study with a large cohort of 70 healthy participants, Spiesshoefer et al. (99) robustly demonstrated that cervical magnetic stimulation effectively assesses peripheral conduction in the diaphragm and inspiratory pathways. They reported minimal variability in DiMEP and compound muscle action potential (CMAP) latencies within and between subjects, in contrast to considerable amplitude variations across sex, age, and side. Furthermore, Zhang et al. (100) showed that magnetic stimulation over the C7 spinous process results in a progressive increase in phrenic nerve conduction time (PNCT) with rising intensity, indicating that such stimulation can effectively depolarize the phrenic nerve to shorten PNCT.

### 2.4 TMS acts on the dorsolateral prefrontal cortex

In contrast to its right hemispheric counterpart, the left dorsolateral prefrontal cortex (DLPFC) plays a critical role in regulating negative affect and generating approach-motivated behaviors (101). Through connections with limbic structures (e.g., the amygdala), it facilitates cognitive reappraisal (reframing emotional stimuli to modulate responses) and inhibits maladaptive emotional reactions (102). Reduced left DLPFC activity constitutes a well-established neural correlate of mood disorders, including depression (101, 103). TMS alleviates anxiety and depression by modulating cortical excitability and neuroplasticity (104, 105), demonstrating its capacity for emotional regulation. This is highly relevant to respiration, as emotional states are known to influence the respiratory center through limbic structures like the amygdala and hypothalamus, often resulting in increased or irregular breathing patterns. It is well established that a bidirectional link exists between respiration and anxiety (106). Therefore, we hypothesize that the emotional benefits conferred by TMS may indirectly lead to the stabilization and improvement of respiratory function.

Patients with CRDs exhibit elevated anxiety and depression levels (107), and up to 74% of patients with COPD experience clinically significant anxiety (108). This comorbidity frequently manifests as insomnia, palpitations, dyspnea, poor sleep quality, and fatigue (109). The basic pathophysiology is defined by a self-perpetuating cycle: dyspnea causes catastrophic interpretation and anticipatory anxiety, leading to activity avoidance, ultimately resulting in additional pulmonary failure and worsening of respiratory distress. The clinical recognition of the bidirectional dysregulation between breathing challenges and anxiety is often described as a vicious cycle (17, 107). TMS may help break this cycle by neuromodulating emotion-processing areas, such as the ACC, leading to enhanced emotional regulation (18). Such emotional regulation may secondarily normalize breathing patterns through a reduced anxiety-mediated respiratory drive (102). Additionally, DLPFC stimulation modulates autonomic balance by enhancing parasympathetic tone and reducing sympathetic dominance (e.g., increased heart rate variability), resulting in respiratory rate deceleration (110). Supporting this, Berger et al. (111) demonstrated that rTMS of the right DLPFC attenuated stress-induced tachycardia and decreased respiratory frequency. Collectively, these mechanisms suggest that TMS may serve as a promising intervention for disrupting the dyspnea–anxiety cycle in patients with CRD through emotional pathway modulation. However, this therapeutic hypothesis warrants further verification through targeted clinical trials.

### 2.5 TMS acts on neurotransmitters

TMS modulates respiratory center excitability through neurotransmitter-mediated mechanisms. In the central nervous system, the regulation of ventilatory drive relies heavily on the dynamics of neurotransmitters. Excitatory neurotransmitters enhance ventilation, whereas inhibitory neurotransmitters decrease respiratory output (112). Key mediators involved in this regulation include glutamate, γ-aminobutyric acid (GABA), glycine, adenosine, serotonin, and dopamine (112–115) many of which also exert parallel effects on cardiovascular function. Notably, glutamate and GABA directly modulate systemic oxygen consumption and carbon dioxide production (114). Experimental evidence has demonstrated that glutamate augments minute ventilation, cardiac output, and metabolic rates (116, 117).

Alterations in GABA (increased inhibition) and glutamate (decreased excitation) levels mediate changes in the central ventilatory drive. Glutamate, the primary excitatory neurotransmitter in the central nervous system, exhibits enhanced transmission efficiency following high-frequency rTMS, increasing cortical excitability and stimulating respiration (118). Conversely, GABA acts as a potent inhibitor of respiratory responses and likely regulates ventilatory drive under both physiological and pathophysiological conditions (119). Michel-Flutot et al. (120) demonstrated that a single session of 10-Hz high-frequency rTMS attenuated hyperexcitability in respiratory pathways via GABA-mediated local inhibition. Similarly, Lenz et al. (121) observed that 10-Hz rTMS reduced postsynaptic GABAergic neurotransmission in neurons. In CRDs, acute and chronic hypoxia alter neurotransmitter concentrations in the brain (122). For example, hypoxia triggers peripheral chemoreceptor activation, which significantly suppresses glutamate release and elevates cerebral GABA levels, thereby promoting ventilatory inhibition (123). Collectively, these findings suggest that TMS modulates the respiratory activity by targeting glutamatergic excitation and GABA inhibition.

Furthermore, studies in both human and animal models have confirmed that rTMS enhances the function of brain-derived neurotrophic factor (BDNF) within the cortex and lymphocytes (124–127). BDNF has been implicated in mediating respiratory plasticity within the PMN pathways (128). In preclinical models, high-frequency rTMS applied to the rat prefrontal cortex increased striatal dopamine release, which in turn mitigated hypoxia-induced respiratory depression (129). By decreasing the neuroinflammatory response, TMS may also cause respiratory plasticity. In total, 10 Hz rTMS treatment alleviates spinal cord (C1–C3) inflammation in rats following C2 hemisection, accelerates neuroplasticity in PMNs, enhances descending respiratory fiber activity in the ventrolateral funiculus, and improves respiratory dysfunction after cervical SCI (130). Although these specific models may not fully represent CRDs, they provide mechanistic evidence that TMS modulates respiratory function through neurotransmitter systems, supporting the feasibility of TMS as an adjunct respiratory therapy.

## 3 The role of TMS in CRDs

### 3.1 Assessment role of TMS in respiration

In an assessment tool to evaluate the integrity of neural conduction pathways, TMS mainly acts as an assessment tool (131, 132). TMS induces electric currents within the cerebral cortex (e.g., the motor cortex) by generating time-varying magnetic fields, thereby activating cortical neurons and eliciting contractions in downstream muscles. These contractions are recorded as motor-evoked potentials (MEPs) via electromyography (EMG), which provides a functional measure of neural conduction from the cortex to the target muscles (133, 134). As previously mentioned, DiMEPs were first evoked using TMS in 1990 (91). Clinically, TMS is employed to assess the central nervous system drive to the diaphragm, enabling the evaluation of corticospinal pathway integrity (61, 135–139). The analysis of MEPs in pathological situations makes it easier to identify the main causes of diaphragmatic dysfunction. For instance, prolonged MEP latency suggests delayed corticospinal conduction, potentially indicating demyelinating lesions or central neuronal injury (59). MEP amplitude indicates decreased motor neuron excitability or axonal loss, usually observed in amyotrophic lateral sclerosis and spinal muscular atrophy (140). Additionally, the calculation of central conduction time, which is defined as the conduction interval from the cortex to the spinal cord, aids in localizing lesions within the central or peripheral neural pathways (141).

Similowski et al. (59) demonstrated that cortical magnetic stimulation evaluated the integrity of the efferent motor pathways from the cortex to the diaphragm, enhancing the diagnosis of respiratory disorders in patients with neurological conditions and assisting in distinguishing between central and peripheral respiratory dysfunctions. Vinit et al. (90) further established TMS as a functional diagnostic tool and therapeutic modality for evaluating synaptic reorganization and neuroplasticity following cervical SCI. DiMEPs serve as quantifiable markers of corticospinal tract integrity, enabling the assessment of respiratory function in patients with SCI or neurodegenerative diseases (142). Additionally, TMS may predict diaphragmatic corticospinal impairment in patients with COPD by assessing diaphragmatic MEP latency and conduction time (135). For instance, in a 2004 study investigating corticospinal pathway excitability in COPD patients, Hopkinson et al. (61) employed TMS to assess diaphragmatic motor thresholds, latencies, and intracortical excitatory/inhibitory circuits. Their findings revealed a significantly prolonged mean phrenic nerve latency, shortened central motor conduction time, and an increased recruitment curve slope for DiMEPs with notably higher amplitudes in the COPD group compared to controls (quadriceps, rectus abdominis, and external oblique muscles). This pattern suggests abnormal corticospinal control of respiratory muscles in COPD patients (15). A analogous investigation in MS reported prolonged DiMEP latency and central motor conduction time (CMCT) by 31% and 23%, respectively, which were less severe than the 76% and 79% prolongations observed in the abductor digiti minimi muscle. The normal phrenic nerve compound muscle action potentials (PN-CMAPs) indicated that the cortico-diaphragmatic pathway is impaired in only a subset of MS patients (143). Taken together, TMS is a valuable tool for evaluating the integrity of the central and peripheral phrenic nerve pathways.

Although TMS provides a noninvasive method for assessing diaphragmatic function through MEP measurements, thereby avoiding the risks associated with traditional invasive techniques, such as phrenic nerve electrical stimulation, its clinical adoption remains limited (144, 145). This limitation stems from two primary factors: the deep anatomical position of the diaphragm necessitates highly sensitive signal acquisition systems, and operator-dependent variables (e.g., target localization accuracy and stimulation intensity selection) significantly influence measurement precision (146, 147). Despite these constraints, TMS has substantial research utility when integrated with complementary modalities, and the integration of functional magnetic resonance imaging facilitates accurate spatial mapping of activation zones in the respiratory cortex. Concurrent application with diaphragmatic ultrasound and EMG permits the simultaneous evaluation of mechanical contractile properties and electrophysiological characteristics (131, 132, 145, 146).

### 3.2 Therapeutic role of TMS in respiration

In clinical practice, a decline in respiratory function arises from multiple contributing factors (148). The most common etiologies include pulmonary and airway diseases and chest wall pathophysiological alterations, as observed in CRDs, such as COPD and interstitial lung disease (ILD) (149). A distinct category comprises neurological dysfunction manifesting as impaired central drive (frequently due to brain disorders) or respiratory muscle weakness (e.g., lesions involving anterior horn cells, peripheral nerves, the neuromuscular junction, or chest wall and diaphragmatic muscles) (150).

Conventional research primarily examines diaphragmatic contraction via TMS to restore respiratory function through the modulation of cortical excitability (31, 64). In a rat model of the phrenic motor circuit, Michel-Flutot et al. (120) pioneered the investigation into the mechanisms by which high-frequency rTMS enhances phrenic motoneuron excitability. By recording DiMEPs to assess excitability, they demonstrated that a single session of 10 Hz rTMS could induce a long-lasting potentiation of phrenic motoneurons—a 59.1 ± 21.1% increase from baseline in DiMEPs—via “local disinhibition” of the GABAergic network. This effect was unique to 10 Hz stimulation and absent in 3 Hz, 30 Hz, and sham stimulation groups, revealing a novel mechanism through which rTMS modulates spinal motoneuron excitability and offering a non-invasive therapeutic avenue for respiratory dysfunction. Furthermore, recent human studies corroborate these benefits. Cao et al. (71) reported that after eight weeks of TMS intervention, post-stroke patients showed significant improvements in ultrasonographic measures of the diaphragm (diaphragmatic thickness; diaphragmatic mobility; diaphragmatic thickening fraction) and pulmonary function indicators (forced vital capacity [FVC], forced expiratory volume in 1 s [FEV1], forced expiratory volume in 1 s/forced vital capacity[FEV1/FVC], peak expiratory flow [PEF], maximum inspiratory pressure [MIP]) on both the affected and unaffected sides. Similarly, in a 60-participant study, Hanan Mohamed et al. found that TMS, whether applied at the cortical or subcortical level and combined with conventional rehabilitation, significantly enhanced diaphragmatic function and daily activity capacity, with cortical TMS yielding superior outcomes. Additionally, beyond common ultrasonographic and pulmonary metrics, Qin Zhang et al. (100) demonstrated that combining external diaphragm pacing (EDP) with repetitive peripheral magnetic stimulation (rPMS) of the phrenic nerve could increase the amplitude of the phrenic nerve CMAP and shorten PNCT. Dan Adler's research identified that cervical magnetic stimulation at 15 Hz and 65% of maximum output represents an optimal parameter set, effectively improving transdiaphragmatic pressure (Pdi) without inducing diaphragmatic fatigue, thereby confirming the therapeutic potential of magnetic stimulation for diaphragmatic conditioning.

In COPD, the etiology of dyspnea extends beyond structural respiratory alterations and encompasses neurological, psychological, and social factors. First, beyond pulmonary parenchymal destruction and airway pathophysiological changes, patients with COPD exhibit diaphragmatic dysfunction (151). Notably, 32%–57% of patients with COPD demonstrate generalized muscle weakness with a concomitant reduction in diaphragmatic strength (152, 153). One major factor is chronic hyperinflation, which induces diaphragmatic remodeling, fiber-type shifting, and ultimate weakness. Second, impaired cortical excitability contributes to diaphragmatic impairment (135). Patients display reduced motor cortex excitability and diminished volitional activation (154). In support of this, Rocha et al. (155) observed decreased gray matter density in the motor cortex (precentral gyrus), indicating attenuated central excitation, which impairs spontaneous activation mechanisms and propagates weakness. Correspondingly, Hopkinson et al. (61) reported significantly prolonged diaphragmatic PMEPL in patients with COPD vs. healthy controls and found that diaphragmatic dysfunction correlated with dyspnea perception severity (155). Contemporary research underscores the multidimensional nature of dyspnea by integrating the sensory–perceptual, affective, and impact domains (19).

Consequently, the respiratory cortical network can be investigated through multiple mechanistic pathways. These include neuromodulatory effects such as GABAergic-mediated disinhibition, as well as serotonergic activation, which promotes the synthesis of brain-derived neurotrophic factor (BDNF). Additional mechanisms involve spinal vascular endothelial growth factor (VEGF) receptor activation, erythropoietin signaling, sustained diaphragmatic inactivation (156–159), and the facilitated release of anti-inflammatory factors (160). Beyond these molecular and cellular pathways, TMS also exhibits dual neuromodulatory effects at the systems level. High-frequency stimulation of the DLPFC enhances amygdala inhibition and attenuates anxiety-related circuit activity. This mood-regulating effect can raise the perceptual threshold for dyspnea and disrupt the dyspnea-anxiety feedback cycle (161, 162). In parallel, adjunctive low-frequency DLPFC stimulation increases parasympathetic tone, thereby promoting respiratory rhythm stability. Taken together, these findings illustrate that TMS modulates respiratory function in a multidimensional manner: it enhances diaphragmatic output via corticospinal pathways while concurrently facilitating neurochemical release and alleviating anxiety-depression components (110). This integrative profile positions TMS as a promising adjuvant therapy for improving respiratory function in patients with chronic respiratory diseases. Nevertheless, given the current scarcity of related clinical studies, these findings warrant cautious interpretation. Furthermore, larger-scale clinical trials are imperative to conclusively establish the efficacy of TMS in treating respiratory dysfunction. Following this detailed discussion of TMS's therapeutic mechanisms and physiological effects on respiration, Table 1 provides a structured overview of the current clinical research landscape regarding TMS applications in the respiratory system.

**Table 1:** Relevant research on transcranial magnetic stimulation in the respiratory system.

Studies	TMS-stimulated region	TMS parameters (type, frequency, intensity)	Outcomes
Murphy et al. (1990) (91)	In the midsagittal line, 1 cm behind the vertex.	PAS, 75%–95% MO	The excitatory route from the motor cortex to the diaphragm has been verified by TMS.
Budzinska et al. (1991) (163)	NA	NA	Alterations in breathing pattern were shown in conscious but highly sedated baboons after TMS
Maskill et al. (1991) (56)	3 cm lateral and 2 cm anterior to Cz (using the 10–20 EEG system)	PAS, 90% MO	1. Unilateral TMS of the motor cortex has shown that diaphragm activation is primarily contralateral. 2. The compound muscle action potentials recorded from the left diaphragm in response to TMS were at their peak when the center of the figure-eight coil was placed approximately 3 cm to the right of the midline and 2–3 cm in front of the auricular plane
Lissens et al. (1994) (83)	2 cm anterior to C3/C (using the 10–20 EEG system)	PAS, 100% MO	There is a direct projection from the motor cortex to the human diaphragm, and transcranial magnetic stimulation (TMS) can activate the diaphragm
Similowski et al. (1996) (57)	Over the vertex	PAS, maximal contraction with maximal stimulator intensity	Cortical magnetic stimulation gives the diaphragm access to the central motor conduction pathway
Zifko et al. (1996) (60)	Over the vertex	PAS, 95% MO	In patients who have CNS involvement, TMS may be used to diagnose and keep an eye on them.
Corfield et al. (1998) (82)	1 cm to the rear of the head vertex	PAS, 65 ± 100% MO	Through the use of TMS, the excitation of the diaphragm's motor cortex is not facilitated by brainstem respiratory neurons, but rather by the corticospinal tract
Khedr et al. (2000) (164)	The midline and 2–3 cm in front of the auricular plane	PAS, 0.3 Hz until the maximum diaphragmatic CMAP was recorded	Using TMS to evaluate DiMEPs, patients with acute stroke have diaphragm dysfunction
Urban et al. (2002) (63)	2–3 cm lateral and 2 cm anterior to the vertex	PAS, 100%–130% of the motor threshold	Conversely, the contralateral motor cortex is mostly responsible for the voluntary activation of the respiratory muscles.
Sharshar et al. (2003) (89)	3 cm forward and 3 cm backward from the vertex	PAS, 100% MO	1. In healthy individuals, voluntary activation of the diaphragm modifies the motor threshold and the stimulus-response curve following TMS of the motor cortex. 2. TMS has been proven to be a powerful experimental tool for studying the spinal cord pathway in the diaphragm
Miscio et al. (2003) (143)	Over the vertex	PAS, 65%–100% MO	In some patients with MS, there is damage to the cortico-diaphragmatic pathway
Demoule et al. (2003) (165)	Over the vertex	PAS, 80%–125% of the motor threshold	TMS may be applied to the study of the diaphragmatic cortical representation in humans in much the same way as for nonrespiratory skeletal muscle
Sharshar et al. (2003) (166)	Intersection of the midsaggital cranial and interaural lines	40 to 100% MO	Using TMS found that supraspinal control of the costal and crural diaphragm is identical during inspiratory tasks
Miscio et al. (2006) (167)	Over the vertex	PAS, 65%–100% MO	The cortical–diaphragm pathway can serve as a method to indicate diaphragm injury
Duguet et al. (2006) (69)	Over the vertex	PAS, 100% MO	TMS can help predict the timeframe in which patients with central respiratory paralysis are likely to regain their ability to breathe independently
Harraf et al. (2008) (168)	2 cm anterior and 4 cm lateral to the vertex	PAS, 140% of the motor threshold	Cortical injury may result in cough in patients with stroke, and it is related to expiratory muscle weakness.
Raux et al. (2010) (95)	SMA	Single-pulse TMS, rTMS 90% MO	The diaphragmatic corticospinal pathway is more excitable in normal humans because of the high-frequency rTMS paradigm over the SMA in normal individuals
Laviolette et al. (2013) (87)	1 cm to the forefront of the site where AH can be induced	cTBS, 110% of the AMT, 3 times per 200 ms, 50 Hz pulse (600 pulses),5 Hz (500 pulses), interval 50 s	SMA can bidirectionally regulate the corticospinal pathway of the diaphragm
Vint et al. (2014) (169)	6 cm from the caudal end of the anterior fontanel	Single-pulse TMS, from 60 to 100% MO	The DiMEPs produced by TMS may be used to assess supraspinal respiratory plasticity and can be successfully recorded in rats
Nierat et al. (2015) (85)	SMA	cTBS,80% of the AMT,200 ms, 3 times, 50 Hz, (600 pulses) 5 Hz (500 pulses), interval 50 s	The 5 Hz pretreatment can decrease ventilation, reduce tidal volume, and shorten inspiratory time
Elnemr et al. (2019) (135)	SMA	PAS, 80% MO	TMS can be used to predict the diaphragmatic corticospinal effect on the diaphragm in patients with COPD
Wang et al. (2019) (136)	C4 (using the 10–20 EEG system)	PAS, 80% MO	Patients who have acute exacerbation of COPD and respiratory failure were more likely to have prolonged CMCT and CMEPL caused by TMS
Michel-Flutot et al. (2021) (120)	6 cm from the caudal end of the anterior fontanel	rTMS, 3 Hz/10 Hz/30 Hz, 30-s intervals delivered at 50% of maximum output	A steady increase in PMN excitability occurs as a result of 10-Hz TMS
Michel-Flutot et al. (2022) (130)	6 cm from the caudal end of the anterior fontanel	Single pulses, 50% MO	After cervical SCI, chronic high-frequency TMS might improve respiratory failure.
Cao et al. (2022) (71)	SMA	rTMS, 5 Hz, 130%of the diaphragm motor threshold on the unaffected side	Following an acute ischemic stroke, TMS can improve pulmonary function

TMS, transcranial magnetic stimulation; rTMS, repetitive TMS; PAS, paired associative stimulation; cTBS, continuous theta burst stimulation; SMA, supplementary motor area; MO, maximum output of the stimulator; AH, abductor hallucis; Cz, central zero; EEG, electroencephalography; CMAP, compound muscle action potential; DiMEPs, diaphragmatic motor-evoked potential; PMN, diaphragm motor neurons; CMCT, central motor conduction time; CMEPL, cortical motor evoked potential latency; MS, multiple sclerosis; COPD, chronic obstructive pulmonary disease; SCI, spinal cord injury; NA, not available. rPMS, repetitive peripheral magnetic stimulation; 6MWT, 6-minute walk test; ADL, activities of daily living; DT, diaphragmatic thickness; DE, diaphragmatic excursion; FVC, forced vital capacity; FEV1, forced expiratory volume in first second; MIP, maximum inspiratory pressure.

### 3.3 The safety of TMS

The international scientific community has evaluated the available evidence on TMS administration in clinical and research settings and has established corresponding safety guidelines (26). A recent review of the safety evidence for repetitive TMS (rTMS) concluded that the procedure is generally well-tolerated by patients, with serious adverse effects being extremely rare—particularly the incidence of seizures, which is very low (170). Potential side effects of TMS include the induction of seizures, transient acute hypomania, syncope, transient headache, neck pain, local discomfort, transient hearing changes, and transient cognitive alterations (29, 171). Supporting this, a meta-analysis of rTMS studies by Slotema et al. (172) reported the following frequencies for the most common adverse events: headache (9.7%), scalp discomfort (9.3%), facial twitching (1.9%), lacrimation (1.5%), local redness (1.3%), and drowsiness (2.5%). In summary, commonly used TMS protocols are safe and exhibit a favorable tolerability profile.

## 4 Discussion

Over the past decade, noninvasive cortical stimulation techniques have advanced significantly, providing powerful tools to enhance our understanding of the intact human brain and enabling innovative, non-pharmacological treatments for neurological and psychiatric disorders. Research on TMS for respiratory diseases and regulation of respiratory function remains exploratory and primarily focuses on evaluating respiratory conduction pathways (26, 31). TMS is a test instrument that must be highly accurate and repeatable. However, the measurement of DiMEPs faces several methodological challenges.

First, precise EMG electrode location is essential for MEP efficacy. During cortically induced muscle contractions, recorded signals represent the summated electrical activity of all muscles beneath the electrodes, raising uncertainty regarding the true diaphragmatic origin (58). Second, the cortical representation of the diaphragm is less accessible than that of other motor regions, yielding smaller MEP amplitudes (150). Third, surface EMG signal acquisition for the diaphragm presents inherent difficulties in ensuring accuracy (60) and consistent noise control across sessions. Clinically, factors such as high body mass index and female sex are associated with greater technical challenges in obtaining reliable diaphragmatic signals. Furthermore, maintaining precise and stable TMS coil positioning over the target cortical area throughout the intervention poses significant practical difficulties. Therefore, accurate targeting and interference mitigation are paramount considerations for both assessment and therapeutic applications. Although technological advances, such as TMS neuronavigation systems, have demonstrated efficacy in reducing these confounders, widespread clinical adoption remains limited by equipment requirements, operational complexity, and operator expertise (173, 174).

Breathing is regulated not only by cortical structures but also by the brainstem (73, 75). When clinical modification of involuntary breathing is required, the brainstem is a logical target. Sharshar et al. (89) demonstrated that TMS stimulation of the motor cortex elicited phrenic MEPs in patients with brain injury, but the responses were significantly attenuated in patients with brainstem lesions. This finding indicates that the corticospinal pathway is dependent on brainstem integrity. However, direct stimulation of brainstem respiratory centers with conventional TMS is technically challenging with conventional TMS due to penetration depth limitations and targeting constraints. Standard clinical TMS systems have a magnetic field penetration depth of approximately 2–3 cm, primarily affecting superficial cortical structures, such as the motor and prefrontal cortices (175). The brainstem lies deep at the skull base (5–6 cm from the scalp), beyond conventional TMS reach (176, 177).

Furthermore, the structural complexity of the brainstem (e.g., the medullary and pontine respiratory centers) necessitates magnetic resonance imaging-guided navigation for precise targeting, which is a technologically demanding requirement (178–180). Although deep TMS or novel approaches offer limited potential, these techniques require higher magnetic field intensities that may exceed established safety thresholds, thereby increasing the risk of seizures or headaches (181). As reviewed by Gandevia et al. (182), respiratory muscles (including the diaphragm) remain under dual control by both the cerebral cortex and brainstem. Consequently, indirect modulation via the cortico-brainstem pathway is a viable alternative. Several studies (150, 169–185) have indicated that motor cortex TMS activates diaphragmatic MEPs and may indirectly influence respiratory muscles through corticospinal pathways rather than direct brainstem action. Given the current technical limitations, this indirect cortico-brainstem modulation strategy is a feasible clinical approach.

Current clinical TMS research predominantly focuses on mental and neurological disorders, with relatively few studies addressing respiratory applications. Long-term follow-up data and large-sample trials are significantly missing. The limited existing clinical investigations have concentrated primarily on respiratory muscle weakness secondary to central nervous system injuries causing central respiratory paralysis. The most recent work on TMS for respiratory dysfunction is by Cao et al. (71), who examined rTMS combined with respiratory muscle training for PR post-ischemic stroke. Their study confirmed that 8 weeks of rTMS (five sessions/week) targeting the diaphragmatic cortical center improved pulmonary function after acute ischemic stroke. However, the absence of DiMEPs measurements precludes the confirmation of the direct diaphragmatic effects of TMS. Substantial preclinical evidence indicates that TMS directly modulates the neuronal membrane potential at the target site and indirectly influences neurotransmitter release, synaptic plasticity, cell survival, and inflammatory or immune processes (31). However, clinical evidence regarding its effect on respiration, particularly in CRDs, remains insufficient.

TMS represents a promising neuromodulatory tool with potential applications in CRDs. We propose five key research priorities to advance the field: (1) Determine if TMS, beyond alleviating prevalent anxiety and depression, can positively modify the clinical course of comorbid CRDs. (2) Elucidate whether TMS alleviates dyspnea by enhancing respiratory motor output, reversing GABAergic depression, or facilitating diaphragmatic function via BDNF pathways. (3) Evaluate if the transient cortical effects of rTMS can be translated into sustained, clinically meaningful benefits for respiratory muscle function in progressive CRDs. (4) DiMEPs as a quantitative tool to localize corticospinal dysfunction and track disease progression. (5) Combine TMS with multimodal techniques (e.g., EEG, fNIRS, neuroimaging) to decode pathological respiratory neural circuitry and guide personalized neuromodulation.

We recommend several interesting research directions for TMS in respiratory dysfunction associated with CRD:(1) The influence of TMS on the emotions of patients with respiratory disorders. TMS regulates anxiety and depression. Anxiety and depression are widespread in CRDs; however, there is no evidence that TMS is effective for anxiety and depression in CRDs. The anxiety of such patients stems from the lung disease itself. (2) The influence of TMS on neurotransmitters. TMS increases the release of neurotransmitters, improves synaptic plasticity, increases cell survival, and alters inflammatory and immune processes. Can the increase in respiratory motor output caused by this change and the adjustment of respiratory depression by GABA and the stimulation of diaphragmatic motor neurons by BDNF alleviate dyspnea in CRDs? (3) The effects of TMS on respiratory muscles and the length of time they last. TMS is effective for diaphragmatic dysfunction caused by neurological injury; however, research on CRDs is lacking. Compared with other devices, rTMS may have an after-effect because it subsequently affects cortical circuits for several minutes to hours. Ultimately, most diseases in these patients progress over time. (4) The use of TMS-MEP to evaluate abnormal conduction of the respiratory corticospinal tract and cortical excitability is more effective in assessing the source of diaphragmatic disorders. (5) TMS combined with EMG: TMS can also be combined with EEG, neuroimaging, near-infrared imaging, and other techniques to further clarify respiratory neural circuits disorders under different physiological and pathological conditions.
